# My Mind Project: the effects of cognitive training for elderly—the study protocol of a prospective randomized intervention study

**DOI:** 10.1007/s40520-016-0570-1

**Published:** 2016-04-22

**Authors:** C. Giuli, P. Fattoretti, C. Gagliardi, E. Mocchegiani, D. Venarucci, M. Balietti, T. Casoli, L. Costarelli, R. Giacconi, M. Malavolta, R. Papa, F. Lattanzio, D. Postacchini

**Affiliations:** 1 Geriatrics Operative Unit, Italian National Research Centre on Aging (INRCA) IRCCS, Contrada Mossa, 63900 Fermo, Italy; 2 Center for Neurobiology of Aging, Italian National Research Centre on Aging (INRCA), via Birarelli, Ancona, Italy; 3 Centre of Socio-Economic Gerontological Research, Italian National Research Centre on Aging (INRCA), via S. Margherita, Ancona, Italy; 4 Nutrition and Aging Centre, Italian National Research Centre on Aging (INRCA), via Birarelli, Ancona, Italy; 5 Biochemical Operative Unit, Italian National Research Centre on Aging (INRCA), Contrada Mossa, Fermo, Italy; 6 Scientific Direction, Italian National Research Centre on Aging (INRCA), via S. Margherita, Ancona, Italy

**Keywords:** Cognitive training, Alzheimer’s disease, Mild cognitive impairment, Elderly people, Biomarkers

## Abstract

**Background:**

Cognitive decline and dementia represent a key problem for public health as they heavily impair social functioning and independent living. The development of new strategies to support recommendations for patients and their caregivers may represent an outstanding step forward.

**Aims:**

To describe the study protocol and methods of “My Mind Project: the effect of cognitive training for elderly” (Grant No. 154/GR-2009-1584108), which investigates, by the use of a multidisciplinary approach, the effects of a comprehensive cognitive training programme on performances in aged subjects with mild–moderate Alzheimer’s disease, mild cognitive impairment and normal cognitive functioning.

**Methods:**

The study is a prospective randomized intervention for the assessment of cognitive training effects in three groups of elderly subjects with different cognitive status. A total of 321 elderly people were enrolled in Marche Region, Italy. Each subject was randomly assigned to an experimental group or to a control group. Cognitive performances and biochemical blood markers have also been analysed before cognitive training (baseline), immediately after termination (follow-up 1), after 6 months (follow-up 2) and after 2 years (follow-up 3).

**Discussion:**

The results will be useful to identify some efficient programmes for the enhancement of cognitive performance in elderly with and without cognitive decline.

**Conclusion:**

The application of a non-pharmacological approach in the treatment of elderly with cognitive disorders could have a profound impact on National Health Service.

## Introduction

Cognitive decline and dementia are pathological conditions that may heavily affect social functioning and independent living. Elderly are particularly susceptible to cognitive diseases, particularly mild cognitive impairment (MCI). Previous studies have evidenced that the annual conversion rates from MCI to Alzheimer’s disease (AD) vary from 5 to 20 % [1], with higher rates in subjects with subjective memory complaints.

To reduce the incidence of these diseases, it is pivotal to identify the treatments that prevent or delay the cognitive decline as well as its progression to dementia. Pharmacological therapies currently available have been proved to be no effective [2], but positive results have been shown by the use of cognitive training, in particular to enhance or regain specific functions, such as memory and attention processes, with a positive impact on subjective well-being, mood status and quality of life of elderly people [3].

The identification of the processes involved in cognitive decline is another key issue, and a multidisciplinary approach might be particularly suitable.

The “My Mind Project” aimed to analyse several aspects related to cognitive performances before and after a cognitive and comprehensive intervention as well as the possible changes in potentially related peripheral biomarkers. Indeed, the variations in cognitive status can be reflected by the modulation of systemic biological parameters. We decided to focalize the attention on the following biomarkers: brain-derived neurotrophic factor (BDNF, lymphocytes mRNA and plasmatic protein), platelet total phospholipase A_2_ (tPLA_2_) activity, the ratio between the levels of two platelet amyloid precursor protein (APP) forms, trace elements, monocyte expression profiling as well as oxidative stress evaluation.

BDNF, a neurotrophin associated with activity-dependent synaptic modulation, is widely expressed in brain structures involved in learning and memory and is also produced by immunocompetent cells [4]. Many studies have taken into account the peripheral blood BDNF levels in AD patients, but data are controversial. Discrepancies are difficult to explain as they might be due to heterogeneity in disease staging [5], not the evaluation of drug use and depressive symptoms [6], underestimation of smoking and alcohol intake impact [7].

PLA_2_ comprises a superfamily of enzymes involved in a wide variety of physiological processes, including phospholipid metabolism, remodelling of cell membranes and intracellular signalling. Cerebral PLA_2_ influences the non-amyloidogenic processing of APP [8], and its functional alterations may have consequences on amyloid-beta peptides (Aβ) production. The use of platelet tPLA_2_ as peripheral biomarker of the neuronal enzyme is peculiarly convincing in the light of the recent finding that *total* PLA_2_ activity in the thrombocytes may specifically mirror the *total* activity in the brain [9].

Aβ, present in the extracellular plaques of AD brain tissue [10], originates by proteolytic processing of APP, a transmembrane protein abundantly expressed in the brain and the peripheral tissues such as platelets. It has been reported that AD platelets show a reduced ratio between the levels of two APP forms, observed as two immunoreactive bands at 130 and 106–110 kDa in Western blotting assays. The APP form ratio allows to detect AD at an early stage and to differentiate it from other types of dementia [11].

Another important aspect regards the role of the immune system in AD pathogenesis. Several evidences support the idea of a brain–immune system breakdown as a potential pathological mechanism in AD [12]. In AD, mononuclear phagocytes (MP) are inefficient [13], and the accumulation of the metabolic end-product Aβ might be due to the failure of the innate immune system. The molecular mechanisms and the gene pathways involved in monocytes deregulation are unknown. Taking into account that also trace elements are essential for optimal immune system function [14], it is increasingly important to understand the process that governs this response in AD. In recent years, it has become evident that trace elements, particularly zinc (Zn), copper (Cu), iron (Fe) and selenium (Se), may play a role in the pathogenesis of AD [15]. Conversely, the possible relationship existing among plasma trace elements levels and performance on cognitive function tests is not well known.

The biological activity of some antioxidant enzymes (superoxide dismutase, glutathione peroxidase, catalase) also contributes to the immune response. In this context, it seems that oxidative stress is mechanistically and chronologically associated with lifestyle-related risk factors for AD and cognitive decline [16]. Indeed, several studies have shown that there is a relationship between lifestyle, healthy dietary patterns and reduced risk of dementia and cognitive decline, as highlighted by all six cross-sectional studies and six of eight longitudinal studies examined in the systematic review of Van de Rest et al. [17]. Physical activity, even moderate, and participation in recreational activities may have a protective effect on mental function and incidence of dementia [18].

The innovative contribution of the My Mind Project (Grant No. 154/GR-2009-1584108 founded by the Italian Ministry of Health and the Marche Region) consists in identifying a comprehensive intervention (training and parameters, both cognitive and biological, useful in monitoring its efficacy) with the scope to develop a multidisciplinary approach to cognitive disease in elderly people. In particular, the use of multidimensional assessment includes the analysis of interrelationships between cognitive, psycho-social and biochemical aspects.

## Methods/design

### Study design

**Fig. 1:**
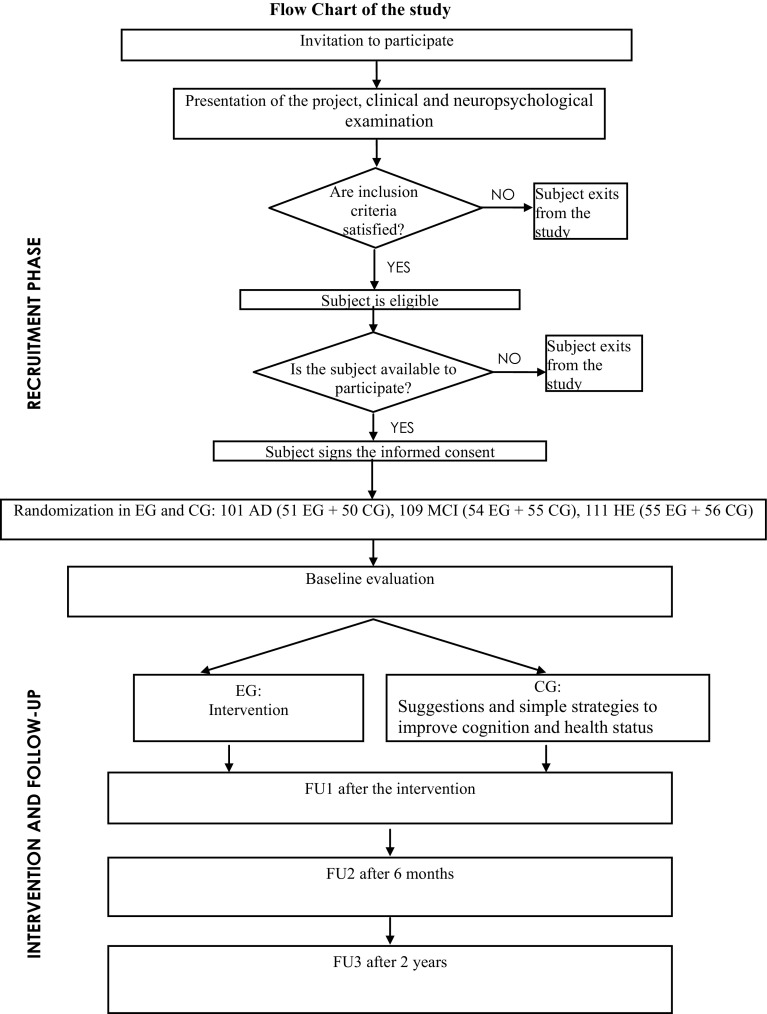
Flow chart of the study

It is a prospective randomized intervention study for the assessment of the effects of cognitive training in three groups of elderly subjects with different cognitive status, using a multidisciplinary approach. The project has a duration of 4 years. Three follow-up phases have been carried out, as shown in Fig. 1

### Recruitment and screening phase

Subjects were enrolled from the Evaluation of Alzheimer’s unit of Geriatrics Operative Unit at the INRCA Hospital in Fermo (Italy) (Fig. 1). The project started on 1 June 2012 and will last until 11 June 2016. Patients were diagnosed with AD or MCI by means of an extended neuropsychological and functional evaluation, neuroimaging and laboratory tests, according to diagnostic guidelines. Healthy elderly (HE) consisted of age-matched individuals who underwent the same clinic and neuropsychological assessment as the other groups. The status of HE was defined as the absence of relevant cognitive diseases.

### Sample

Three hundred and twenty-one community-dwelling elderly subjects (aged ≥65 years) living in Marche Region were enrolled and divided into three groups, according to their cognitive status: 111 subjects without relevant cognitive diseases (HE), 109 subjects with MCI and 101 subjects affected by mild–moderate AD.

### Inclusion and exclusion criteria

Eligibility has been determined after a complete medical and neuropsychological examination to assess the inclusion/exclusion criteria.

The inclusion criteria included (a) subjects aged 65 years or older, (b) availability during the training and testing phases, (c) presence of a caregiver for subjects with cognitive decline (AD and MCI subjects).

Exclusion criteria included (a) the presence of serious medical and psychiatric conditions, and sensori-motor deficits that would prevent the participation in the training; (b) participation in previous cognitive training; (c) subjects with severe AD; (d) the presence of neurodegenerative disorders different from AD.

### Randomization

After the enrolment, the subjects have been assigned to the experimental group (EG) or the control group (CG) and performed a first evaluation, as shown in Fig. 1.

### Sample size calculation

It was based on the null hypothesis that proportion of “success” and “failure” to the intervention is equal in the two groups of subjects (i.e. EG and CG), such as OR = 1.

Success and failure were defined differently for each group of subjects (i.e. HE, MCI, AD) on the basis of a significant variation in the primary outcomes (see below).

Considering an OR = 1.5 indicative of an effect of cognitive training, a total sample of 300 subjects (i.e. 100 subjects per group) allow to reach a 90 % of statistical power using logistic regression at the 5 % level (two-tailed). Drop-out rate was set at 20 %; indeed, a total of 321 subjects were recruited.

### Primary outcome: successful to cognitive training

The effect of training has been measured by evaluating some specific cognitive indicators after the application of effective mnemonics and techniques on cognitive performance.

The study involved the evaluation of subjects who succeed positively to cognitive training. Each group was differently assessed, choosing different outcomes [19, 20].

The first outcome to evaluate the enhancement of cognitive performance in subjects with AD was calculated using ADAS [21]. A decrease in the total score of ADAS of at least two scores measured after the cognitive intervention was an indicative of the enhancement of cognitive performance.

For HE, the first outcome was calculated using the “List of Words” [25]. An increase of 1.5 words in this test measured after the intervention was an indicative of the enhancement of cognitive performance. For MCI subjects, an increase in score of 0.5 of “Word Pairs Learning Test” [27], was taken into account.

### Secondary outcomes

They include

- The assessment of the effects of intervention and the relationship between psychological status, lifestyle characteristics, functional and health status, biochemical aspects by the use of a multidisciplinary approach.
- The investigation of rate of percentage of subjects with MCI who converted to dementia.
- The identification of biochemical markers correlated with the cognitive training in HE, MCI, AD.

### Neuropsychological assessment before and after cognitive training

A comprehensive neuropsychological test battery was conducted in all participants at pretest (baseline) and at the three phases of follow-up. The assessment of cognitive and psychological status included many instruments, as indicated in Table 1.

**Table 1:** List of assessment instruments by group

Instruments	HE	MCI	AD
Overall cognitive status			
MMSE [22]	^a^	^a^	^a^
Memory			
Supra-span of Corsi [23]	n.a.	^a^	^a^
Backward and forward verbal span [24, 25]	^a^	^a^	^a^
List of words [25]	^a^	n.a.	n.a.
Prose memory test [26]	n.a.	^a^	^a^
Word pairing learning test [27]	n.a.	^a^	^a^
Attention			
Attentive matrices [26]	^a^	^a^	^a^
Language and executive functions			
SVF and PVF [28]	^a^	^a^	^a^
Cognitive decline in dementia			
ADAS [29]	n.a.	n.a.	^a^
Overall severity			
CDR [30]	n.a.	^a^	^a^
Psychological assessment			
GDS-30 [31]	^a^	^a^	^a^
PSS [32]	^a^	^a^	n.a.
Questionnaire of well-being [33]	^a^	^a^	n.a.
MAC-Q [34]	^a^	^a^	n.a.
Questionnaire of confidence [33]	^a^	n.a.	n.a.
Functional status			
ADL [35]	^a^	^a^	^a^
IADL [36]	^a^	^a^	^a^
Social networks			
LSNS [37]	^a^	^a^	^a^
Lifestyle			
Lifestyle questionnaire	^a^	^a^	^a^
PASE [38]	^a^	^a^	^a^

*n.a.* not applicable for this group

^a^Applicable for this group

### Intervention

The programme lasted about 2 months for each subject. The main aim of the intervention was to improve different cognitive functions, in order to activate and to motivate participants to ameliorate their cognitive health behaviour by remaining cognitively active and compensating deficits with learned mnemonic strategies after training.

Different comprehensive training methods have been applied to the different groups of subjects, on the basis of their cognitive status. We focalized the intervention not only to cognitive enhancement, but also on many aspects such as advice and psycho-education about healthy lifestyle strategies to maintain cognitive reserves and engagement in leisure activities. Training has been applied for subjects with MCI and with mild–moderate AD using an individualized approach to help them to identify their individual goals and practise strategies focused on them.

The intervention for HE consisted of 10 sessions of 90 min in groups of about 10 participants, once a week. The LAB-I methodology [33], which includes the learning of some effective mnemonics and techniques (such as visual imagery, creation of stories, mental associations, remembering word lists and sequences of items), was used. The addressed domains were, in particular, working memory and learning processes. According to the latter methodology that uses a metacognitive and motivational approach, the participants were also required to perform at-home exercises each day prior to the subsequent session. Another important aim of this enhancement training is focused on maximizing social participation.

MCI group received an individual comprehensive multi-modal training of 10 individual sessions of 45 min, once a week, which included psycho-education about memory loss, and restorative and compensatory cognitive training, which consists of learning strategies for orientation, memory, categorization and clustering. Since MCI subjects often present level of anxiety, stress and/or depression, due to their consciousness of cognitive decline, the effect of metacognition was evaluated to analyse their role on cognitive performances.

AD group received a comprehensive intervention of 10 individual sessions of 45 min each, once a week, including restorative cognitive training addressed in particular to the empowerment of attention functions, orientation, planning of activities of daily living and episodic and prospective memory. Another aim of intervention was to decrease functional disability maximizing engagement in activities of daily living and healthy lifestyle and to support patients and their caregivers for psychological disorders. Participants were also asked to perform homework exercises each day with the help and support of a caregiver.

### Blood sample collections

**Fig. 2:**
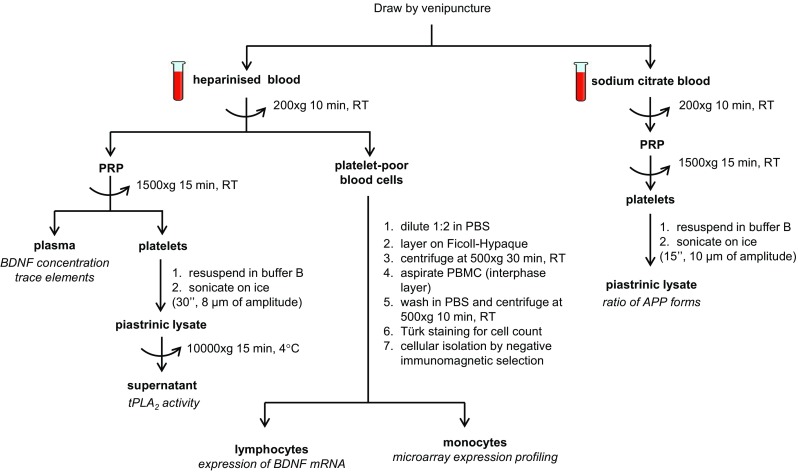
Schematic of blood processing. Composition of buffer B: 10 mM Tris-HCl pH 7.4, 10 mM EDTA, 0.1 mM PMSF, 0.01 mg/l aprotinin and 0.01 mg/l leupeptin. Density of Ficoll-Hypaque = 1.077 g/ml. Storage temperature of isolated samples = −80 °C. *PRP* platelet-rich plasma, *PBMC* peripheral blood mononuclear cell, *RT* room temperature

Blood is drawn from the cubital vein between 8:00 and 9:00 AM, in fasting state. A total of thirty millilitres of blood was drawn from each individual at baseline and at each phase of FU. The blood is divided as shown in Fig. 2. Plasma cortisol, ACTH, vitamin B12, folic acid, albumin and creatinine have been assessed.

### tPLA_2_ activity determination

tPLA_2_ enzymatic activity is determined by a commercial kit (cPLA_2_ Assay Kit, Cayman Chemical Company, Michigan, USA), normalized by protein concentration and expressed as nmol/min/mg. Since samples are not preliminarily purified for sPLA_2_ or treated by iPLA_2_-specific inhibitors, the data obtained can be referred to tPLA_2_. All samples are measured in duplicate. Protein concentration is determined by the Lowry method [39].

### mRNA BDNF determination (qRT-PCR)

Peripheral blood mononuclear cells (PBMCs) are isolated by conventional density gradient centrifugation, and the lymphocytes are separated from the monocytes using Dynabeads Untouched Human Monocytes kit (Invitrogen, USA). This determination method is shown in Casoli et al. [19].

### Plasma BDNF assay

Plasma levels of BDNF are measured using commercial enzyme-linked immunosorbent assay (ELISA) method (BDNF Human ELISA kit, ABCAM, Cambridge, UK), according to the manufacturer’s instructions. All samples and standards are measured in duplicate, and the means of the duplicate are used for statistical analyses. The detection limit for BDNF is typically less than 80 pg/ml.

### Ratio between platelet APP forms

The samples were separated on 7.5 % SDS–PAGE and electrotransferred to nitrocellulose membranes. After blocking with 5 % non-fat dry milk, the membranes were incubated with monoclonal 22C11, diluted 1:1000 (Becton–Dickinson). Blots were visualized using the enhanced chemiluminescence detection kit (Amersham Biosciences). The results are expressed as the ratio between the optical density of the upper (130-kDa) and the lower (106- to 110-kDa) APP immunoreactive bands using β-actin as the internal standard.

### Oxidative stress level

For the determination of oxidative stress, the d-ROMs test (Diacron, Grosseto, Italy) has been used. It is based on Fenton’s reaction, and the values will be expressed as equivalent units (0.08 mg/dl) evaluated by means of free radicals and antioxidant systems (FRAS, Diacron, Grosseto, Italy).

### Trace elements determinations in human plasma

Plasma zinc (Zn), copper (Cu), iron (Fe) and selenium (Se) levels are determined by a Thermo XII Series ICP-MS device (Thermo Electron Corporation, Waltham, MA, USA) following the method previously reported [40].

### Monocyte isolation, RNA extraction, expression profiling and data analysis

Total RNA was extracted from the monocytes using the RNeasy Mini Kit (Qiagen, Hilden, Germany) as recommended by the manufacturer. Purified RNA was quantified using a NanoDrop spectrophotometer, and RNA integrity was determined by gel electrophoresis. The microarray procedure was performed according to the Affymetrix protocols (Santa Clara, CA, USA). Hybridization cocktails containing fragmented, end-labelled single-stranded cDNA were prepared and hybridized to GeneChip Human Exon 1.0 ST arrays (Affymetrix, Santa Clara, CA, USA). Washing and scanning were performed using GeneChip^®^ Fluidics Station 450 and GeneChip^®^ Scanner 3000 7G (Affymetrix Inc.) and converted to numerical data using GeneChip^®^ Command Console^®^ Software (AGCC). The microarray data analysis will be performed by Partek Genomics Solution 6.6 software (http://www.partek.com). The robust multi-array analysis (RMA) of Irizarry et al.’s algorithm will be used for probe set (exon-level) intensity analysis. Exon-level data will then be filtered to include only those probe sets that are in the “core” meta-probe list, which represents 17 800 RefSeq genes and full-length GenBank mRNAs. Within this gene set, the analysis of variance (ANOVA) and multi-test correction for *P* values in Partek Genomic Suite will be used to identify alternative splicing events. A list of genes with significant alternative spliced events will be generated by using a 0.05 FDR criterion as a significant cut-off. The genes will be sorted based on gene function using Partek Pathway software.

### Statistical analysis

Analysis will be carried out by the software SPSS 16 (SPSS Inc. Chicago, Illinois), separately for each group of subjects and across groups.

Descriptive analysis will be used to check the distribution of the data. Bivariate analysis will be used to compare within each group the characteristics of experimental and control groups, by chi-square for frequencies and *t* test for continuous variables. Paired tests will be used to check for the variation in cognitive measures before and after intervention within each group. General linear models will be used to verify the effect of intervention in the outcome measures, adjusting for covariates.

Each subject will be classified on the basis of primary outcomes and then included in multiple logistic regression models to identify the predictors of improvements in cognitive status, adjusting for covariates. Repeated-measures ANOVA will be used to examine the training effect on biochemical parameters adjusted for age and sex. The significance level will be set at *P* < 0.05.

## Discussion

This project is designed to investigate the effects of cognitive training on different groups of elderly people. As many studies indicated that non-pharmacological strategies could be an effective intervention for elderly people with and without cognitive decline [41, 42], we choose to identify the effects of interventions of cognitive training, using a multidisciplinary point of view.

The results of this project could support and have a positive impact on National Health Service in order to produce new knowledge on cognitive decline. Surely, the analysis of the relationship between clinical, biochemical and lifestyle characteristics of elderly subjects represents an integrated approach for the study of cognitive decline. Moreover, few studies compared elderly subjects with different cognitive status using a multidisciplinary approach including the analysis of biomarkers.

Previous studies evidenced the importance of identification of rehabilitation model to guide the assessment of interventions in elderly people with cognitive deficit and recognized the usefulness of a comprehensive multi-modal cognitive training, also based on lifestyle intervention and psychological support [43].

Indeed, the preliminary identification and treatment for MCI and dementia could permit to intervene by means of a non-pharmacological approach. The characteristics of subjects who obtained a success to cognitive training will be analysed in detail, taking into account also the role of biomarkers and psycho-social aspects. MCI subjects could be treated preliminarily with this intervention in order to slow the rate of conversion into dementia, with a consequent positive effect on well-being, mood status and perceived stress level. In this way, it is possible to reduce the costs related to dementia and care-giving, using primary and secondary prevention of the cognitive decline, also reducing or delaying the degree of disability and functionality. Learning mnemonic strategies used for the enhancement of cognitive functions could be a strong benefit for the elderly, to increase personal self-esteem, to live independently for a longer period of time and to allow their caregivers a better care management.

Finally, the multidisciplinary approach and the use of multidimensional assessment could allow us to determine the best way to provide an optimal management of cognitive decline. The findings of this study will provide both healthcare professionals and elderly people with an accurate and detailed knowledge of the aspects related to cognitive decline. So, it will be possible to identify some nutritional, biological and lifestyle factors that could be associated with better cognitive performance. The analysis of biomarkers involved in the onset of cognitive decline will permit the identification of the factors related to cognitive impairment and dementia. In this way, some health programmes aimed at preventing cognitive decline and/or dementia through specific interventions of healthy lifestyle and prevention strategies will be outlined on the basis of obtained findings.
